# 

**Published:** 1885-05

**Authors:** 


					﻿Vol. VI.
MAY, -1-8^5.
No. 5.
THE
IntodentPraelitioBtr:
A	JOURNAL
DEVOTED TO
DENTAL AND ORAL SCIENCE.
W. C. BARRETT, M. D., D. D. S.,
EDITOR.
The Hew York Dental Journal Association,
PUBLISHERS,
No. 35 West 46th Street, NTew York.
AND
11 W. Chippewa St., Buffalo, NT. Y.
$2.50 Per Annum in Advance, .... Single Copies, 25 Cents.
Entered at the Post-Office, Buffalo, N. Y., as Second-Class matter.
				

